# COVID-19 pandemic: Ophthalmic practice and precautions in a tertiary eye hospital in Iran

**DOI:** 10.1017/ice.2020.164

**Published:** 2020-04-23

**Authors:** Zahra Moravvej, Reza Soltani-Moghadam, Azam Ahmadian Yazdi, Kianoush Shahraki

**Affiliations:** 1 Eye Research Center, Eye Department, Amiralmomenin Hospital, School of Medicine, Guilan University of Medical Sciences, Rasht, Iran; 2 Department of Oral and Maxillofacial Radiology, School of Dentistry, Mashhad University of Medical Sciences, Mashhad, Iran; 3 Infection Control Committee of Oral and Maxillofacial Radiology Department, School of Dentistry, Mashhad University of Medical Sciences, Mashhad, Iran; 4 Eye Research Center, Farabi Eye Hospital, Tehran University of Medical Sciences, Iran

*To the Editor*—The novel coronavirus 2019 (SARS-CoV-2) outbreak has reached a critical state, and >200 countries worldwide have been affected. Iran was among the first countries that encountered the virus at a nationwide threat level.^1^ The pandemic has imposed numerous burdens on society and the healthcare system.

Medical specialties involve various examinations that may put patients and practitioners at risk of infection. Ophthalmic instruments may act as medium for viral transmission and ophthalmic healthcare facilities pose a risk of cross infection.^2,3^ A recent review suggested that as coronaviruses are able to develop a wide range of ocular manifestations; thus, ophthalmologists should be cautious to prevent possible transmission through ocular tissue.^4^ Cases of conjunctivitis have been reported in COVID-19 patients.^5,6^ In one report, 2 patients tested positive for SARS-CoV-2 using reverse‐transcription polymerase chain reaction (rRT‐PCR) assay of conjunctival secretions.^5^ Moreover, the nasolacrimal duct may act as a pathway to transfer the virus from the eye to the nasopharynx.^7^

Here, we address the prevention strategies employed against COVID-19 according to assessments of infection control experts and ophthalmologists, in Amiralmomenin Hospital a tertiary referral eye hospital in Guilan, Iran. To the best of our knowledge, this is the second report to describe the actions employed in an ophthalmology hospital setting.^8^ At our institution preventive measures were applied in 3 main aspects outlined below.

## Patient management and screening

The appointments of patients needing ophthalmic examinations (including those who had undergone corneal graft, cataract and vitreoretinal surgeries) were rearranged and other nonurgent appointments were deferred. Patients with a medical history of immune-suppression were appointed to be examined on a specific day. All elective ophthalmic surgeries and procedures were postponed, and patients were notified in advance; however, urgent ocular operations continued as normal.

The daily number of patient referrals to the ophthalmic emergency unit decreased at an average of 58% compared to the previous month’s daily average. This decrease was due to the hospital’s relational unit advising local residents to seek ophthalmic care only when essential.

Patients referring to the emergency unit were screened at the point of entry. The ophthalmic complaint was evaluated at triage by an eye-care nurse, and nonemergent conditions were requested to return for examination after the outbreak resolved. If the patient was physically capable, the companion would be asked to wait outside. All patients were asked about any related symptoms and underwent temperature screening. According to WHO definitions,^9^ suspected COVID-19 cases would be isolated and transferred to a COVID-19 referral center for further evaluation.

A safe distance (1.5 m) was assured between patients who were required to sit in the waiting room. Effort was made to maintain minimum waiting and consultation time. To avoid redundant visits to the hospital, patients who were managed in an outpatient setting were contacted via phone by eye-care professionals at appropriate intervals. Those who needed inpatient care (eg, open globe injuries, orbital cellulitis) were hospitalized, and separate single rooms were assigned in the ward. Hospitalized patients were checked for symptoms of COVID-19 and fever on a regular daily basis.

## Equipment and surface disinfection

Environmental surfaces frequently touched by staff and patients, such as light switches, door knobs, and nursing stations were cleaned according to Centers for Disease Control and Prevention (CDC) recommendations.^10^ The examination and waiting room floor were cleaned on a 4-hour routine with suitable disinfectants (eg, sodium hypochlorite, 70% ethanol, or an alternative disinfectant). After each patient left the room, the equipment used, including the chin and head rests of the slit-lamp, direct and indirect ophthalmoscope, were cleaned with equipment detergent. Instruments that came into contact with the patient, such as contact lenses and ultrasound probes were cleaned according to the manufacturer’s instructions. The Goldmann applanation tonometer’s head piece was cleaned with 70% ethanol or hydrogen peroxide 3% before and after use. The aforementioned instruments were rinsed under running water to remove disinfectants and to prevent damage to the cornea.

## Personnel and physician protection

Healthcare personnel were asked to refrain from leaving the province. Personnel were also monitored for any signs of SARS-CoV-2 infection at the beginning of every shift. Healthcare providers with suspicious symptoms stopped working and were given a 14-day leave of absence. They were isolated and they sought medical treatment according to their condition.

The personal protective equipment for the eye-care nursing staff, ophthalmology residents, and attending eye surgeons included Latex gloves, eye protection (goggles or face shields), a surgical-style face mask, a long-sleeved fluid-resistant gown, and disposable shoe covers. Prepacked sets including the aforementioned equipment were prepared for each individual and were delivered at the beginning of every shift. As advised by the European Society for Cataract and Refractive Surgery (ESCRS), protective shields were installed on slit-lamps.

The ophthalmologists used single-use cotton-swab applicators during examination to avoid touching the patient’s face and eyelid. Hand washing was mandatory between each patient. Gloves were disposed after contact with the patient and hand washing with an alcohol‐based antiseptic was performed subsequently.

In the midst of this crisis, hospitals continue to face shortages of personal protective equipment. We designated special safety measures including appropriate hand washing technique between each patient; other practices, such as the extended use of face masks, were also instructed. Transparency films were cut out to make face shields and plastic bags were used as shoe covers. The number of staff was also limited. These management strategies helped overcome the shortage and limit the spread of the virus.

The experience and preventive strategies mentioned should be of help in similar ophthalmic or subspecialty healthcare facilities. Ophthalmologists and local infection control teams should consider the regional viral extent when applying preventive measures. Hopefully, appropriate precautions will shorten the pandemic period and benefit the whole world.
